# Intraluminal pressure patterns in the human colon assessed by high-resolution manometry

**DOI:** 10.1038/srep41436

**Published:** 2017-02-20

**Authors:** Ji-Hong Chen, Yuanjie Yu, Zixian Yang, Wen-Zhen Yu, Wu Lan Chen, Hui Yu, Marie Jeong-Min Kim, Min Huang, Shiyun Tan, Hesheng Luo, Jianfeng Chen, Jiande D. Z. Chen, Jan D. Huizinga

**Affiliations:** 1 Renmin Hospital of Wuhan University, Key Laboratory of Hubei Province for Digestive System Diseases, Wuhan, Hubei, China; 2 McMaster University, Farncombe Family Digestive Health Research Institute, Department of Medicine, Division of Gastroenterology, Hamilton, Ontario, Canada; 3 Honours Biology Pharmacology Coop Program, McMaster University, Hamilton, Canada; 4 Honours Health Sciences Program, McMaster University, Hamilton, Canada; 5 MedKinetic Inc., Ningbo, China; 6 Johns Hopkins University School of Medicine, Baltimore, USA

## Abstract

Assessment of colonic motor dysfunction is rarely done because of inadequate methodology and lack of knowledge about normal motor patterns. Here we report on elucidation of intraluminal pressure patterns using High Resolution Colonic Manometry during a baseline period and in response to a meal, in 15 patients with constipation, chronically dependent on laxatives, 5 healthy volunteers and 9 patients with minor, transient, IBS-like symptoms but no sign of constipation. Simultaneous pressure waves (SPWs) were the most prominent propulsive motor pattern, associated with gas expulsion and anal sphincter relaxation, inferred to be associated with fast propagating contractions. Isolated pressure transients occurred in most sensors, ranging in amplitude from 5–230 mmHg. Rhythmic haustral boundary pressure transients occurred at sensors about 4–5 cm apart. Synchronized haustral pressure waves, covering 3–5 cm of the colon occurred to create a characteristic intrahaustral cyclic motor pattern at 3–6 cycles/min, propagating in mixed direction. This activity abruptly alternated with erratic patterns resembling the segmentation motor pattern of the small intestine. High amplitude propagating pressure waves (HAPWs) were too rare to contribute to function assessment in most subjects. Most patients, dependent on laxatives for defecation, were able to generate normal motor patterns in response to a meal.

The human colon is one of the least understood organs of the body despite the fact that many common disorders such as chronic constipation or IBS are related to abnormal colon function^1^. These functional bowel disorders are a source of marked reduction in quality of life of those affected and add significant costs to the health care system of every country^2^,^3^,^4^,^5^,^6^,^7^. Although chronic constipation in adults is assumed to be the result of colonic motor dysfunction, investigations into human colonic motility is rarely done because of real and perceived technical complexities and difficulties with interpretation of motility recordings. Although radio-opaque markers^8^ and the SmartPill^®^ can measure colonic transit, they do not give information about motor patterns that underlie peristalsis or mixing and absorption. Important knowledge has been gained from 24-hour colon manometry^9^,^10^; however, traditional colonic manometry^11^,^12^ is generally of low resolution because the sensors are far apart; any activity between recording sites must be assumed, providing potentially erroneous information^13^,^14^. Recently, high-resolution manometry has entered the field with promising results^3^; the spacing between sensors is decreased to 1 cm and thus recordings give a much more comprehensive assessment of motor patterns. All motor patterns can be more accurately resolved and used to potentially identify motor dysfunction^13^,^15^,^16^. However, our understanding of the full spectrum of colonic pressure and motor patterns, which is essential to evaluate colonic motor dysfunction, is still in its infancy.

Patients with chronic constipation are often not sufficiently helped by treatments provided; their quality of life frequently remains suboptimal and surgery may be considered. The effects on children and their families can be devastating. In order to evaluate if High Resolution Colonic Manometry (HRCM) can help diagnose motor dysfunction in these patients and help make better decisions about individual management, first a full understanding of colonic motor activities is needed. Our objectives were to identify and characterize all motor patterns in patients with chronic constipation and control subjects, to identify potentially unique motor patterns in our patients, and to design management based on the HRCM results. The present report deals primarily with the first two objectives. We performed HRCM in 15 patients with chronic constipation according to Rome III criteria, dependent on laxatives for defecation, and in two control groups: healthy volunteers and patients with IBS-like symptoms of short duration and without pain and without any sign of constipation. We used a solid-state catheter with 36 sensors spaced 1 cm apart. We extensively analyzed all pressure patterns with a focus on simultaneous pressure waves and isolated pressure transients. Although these motor patterns have thus far received little attention, we predict that they will become important for understanding normal colon motility, and for diagnosis and management of colonic motor dysfunction.

## Results

The main objective of this study was the identification of motor patterns in the human colon; hence, the motor patterns observed will be described based on all subjects studied, followed by data comparing motor patterns in the different subject groups.

### Subject characteristics

Fifteen patients with severe chronic constipation, according to Rome III criteria, varying in duration from 3 to 25 years were enrolled in the study. All 15 patients depended on stimulant laxatives (Senna or Chinese rhubarb) for defecation for more than one year; all had normal colonoscopy and normal anorectal manometry including the balloon expulsion test, ruling out outlet obstruction. The constipation group consisted of 13 females, age ranging from 27–57 (average 44.9) and 2 males ages 44 and 49. Two control groups were studied. One group consisted of 5 healthy volunteers who did not have any bowel complaints, and were not on any medication that could be expected to have an effect on bowel function; this group consisted of 3 females and 2 males, ranging from 29 to 58 years (average 42.6). Another group consisted of 9 patients who presented with minor transient IBS-like symptoms without pain (see methods for further information); this patient control group consisted of 9 males, age ranging from 25–50 years (average 39.0).

### Simultaneous pressure waves (SPWs)

**Figure 1:**
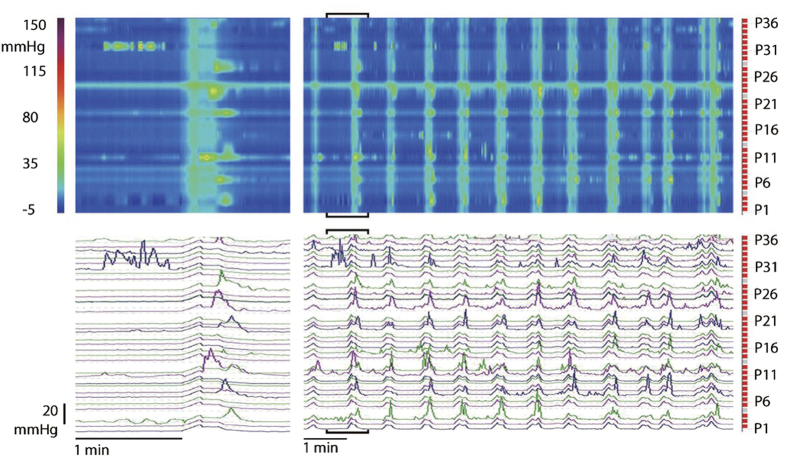
Rhythmic Simultaneous Pressure Waves (SPWs). Left panels: a typical SPW is shown in a spatiotemporal map (35 cm × 2 min) with the associated pressure traces below. Typically, an SPW has superimposed pressure peaks on the downward slope. The SPW is an enlargement of the bracketed area in the right panel. Right panels: a 10 min section shows highly rhythmic SPWs at baseline. From a patient with chronic constipation; sensor P36 is 85 cm from anal verge.

**Figure 2:**
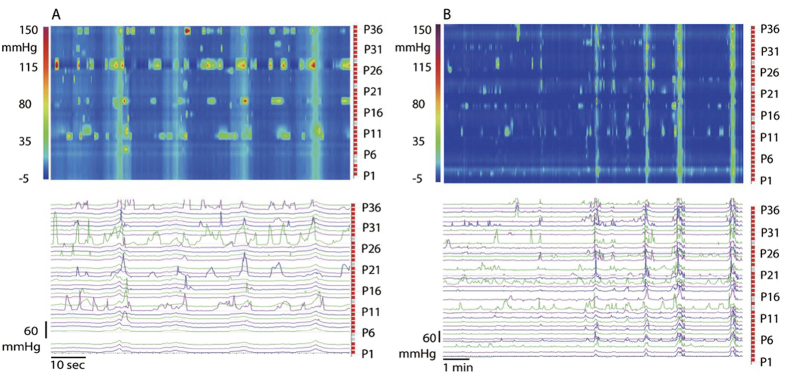
Simultaneous Pressure Waves and Isolated Pressure Transients. (**A**) Four SPWs of relatively low amplitude with high amplitude haustral boundary pressure transients. Pressure transients were prominent in sensors 11,19, 27 and 35, which is 8 cm apart. Such a pattern (prominent in sensors 4–8 cm apart) was very common and strongly suggestive of being associated with haustral boundary contractions. The SPWs occurred rhythmically at 2 cycles/min. All four SPWs were associated with gas expulsion. From a control subject with sensor P36 at 45 cm from the anal verge. (**B**) Four SPWs of increasing amplitude, reflected in the spatiotemporal map as well as in the pressure traces. The last three SPWs were associated with gas expulsion. Scattered isolated pressure transients are present. From a patient with constipation; sensor P36 is 40 cm from anal verge.

SPWs were transient increases in intraluminal pressure, most often covering the entire 35 cm of the colon assessed (Figs 1,2). A common appearance was a relatively fast rate of rise up to ~20 mmHg in ~ 8 s, and a much slower irregular rate of return to baseline over ~12 s with superimposed pressure transients (Figs 1,2).

Considering all subjects, SPWs occurred at a frequency of 12.4 ± 9.6 SPWs per 30 min (range 0–41) at baseline, and 16.2 ± 14.0 SPWs per 30 min (range 0–62) (t = −1.9, df = 28, P = 0.07) after the meal (n = 29). The maximal frequency (see methods) was 1.3 ± 0.5 cycles/min (range 0.7–2.5) at baseline, and 1.8 ± 0.7 cycles/min (range 0.3–3.0) (t = −3.5, df = 18, P = 0.003) after the meal. The SPW amplitude was 14.4 ± 6.1 mmHg (range 6.0–30.0) at baseline, and 13.6 ± 8.7 mmHg (range 2.8–49.0) (t = 0.2, df = 25, P = 0.84) after the meal.

The SPWs spanned 33.1 ± 5.0 cm (range 14–35) at baseline, and 29.7 ± 6.6 cm (range 16–35 cm) (t = 3.0, df = 24, P = 0.007) after the meal. The SPW duration was 15.4 ± 4.5 s (range 3.2–24.0) at baseline, and 14.1 ± 4.2 (range 2.8–22.9) after the meal (t = 2.9, df = 24, P = 0.009).

**Figure 3:**
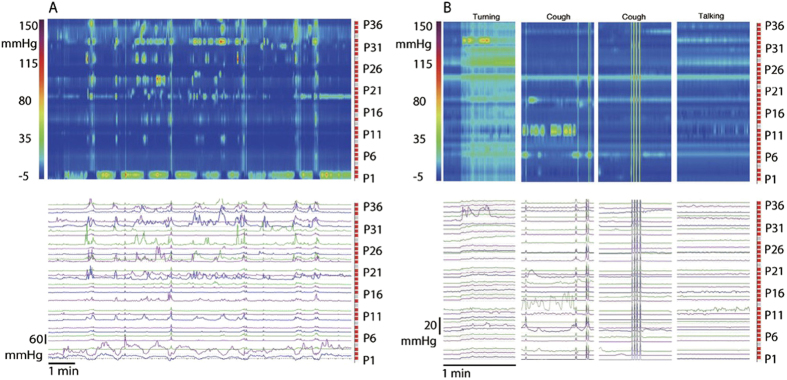
SPWs, internal anal sphincter relaxation and artifactual pressurization. (**A**) Rhythmic SPW activity at 1 cycle/min of variable amplitude. The internal anal sphincter relaxes upon an approaching SPW, even those of relatively low amplitude. The relaxation can be seen to precede the arrival of the SPW. From a patient with constipation; sensor P1 and P2 are at the internal anal sphincter. A cough at ~2.5 min leads to a simultaneous pressure change but is not associated with sphincter relaxation. The last two SPWs were associated with gas expulsion. (**B**) Identification of artifacts due to abdominal pressure changes. Turning of the body from one side to the other causes marked changes in abdominal and intraluminal pressure and has to be noted at all times during the motor function test. Coughs cause sharp simultaneous pressure increases not resembling in any way the SPWs. Talking rarely gives noticeable pressure changes. All data are from a patient with constipation; sensor P36 is 85 cm from anal verge.

After the meal response was evaluated, the catheter was partially pulled out, just enough to capture the internal anal sphincter pressure (n = 3). SPWs upon approaching the internal anal sphincter were associated with internal anal sphincter relaxation, even if the SPWs were of low amplitude, evaluated for 32 SPWs (Fig. 3A). Subjects were asked to always allow gas to be expelled without straining or attempts to hold it back. Nine of 32 SPWs were associated with gas expulsion, in an otherwise empty colon; they were not associated with urge to defecate. In all subjects studied, without exception, when the patient reported gas expulsion, an SPW occurred.

### Exclusion of artifacts

An increase in intraluminal pressure can occur with an increase in abdominal pressure, hence it was important to distinguish changes in intraluminal pressure due to abdominal pressure increases from those due to contractions of the colonic musculature (Fig. 3B). Coughs caused short sharp increases in pressure (Fig. 3B). Body shifts were identified as non-distinct low amplitude changes in pressure in all sensors for the duration of the body movements and were often associated with sustained (non-rhythmic) increases in pressure at single sensors (Fig. 3B). Luminal pressurizations caused by coughing or body movements were associated with an increase in internal anal sphincter pressure, not with sphincter relaxation. Talking did not always lead to a change in abdominal pressure and caused low amplitude increases of about 5 mmHg when it did. Drinking sometimes causes a short increase in pressure of about 10 mmHg throughout the colon, while no change was seen at other times. All events reported by the patient and/or observed by investigators were noted. All SPWs identified in the present study occurred without any visible cues that could potentially reflect a change in abdominal pressure.

### Comparison of SPWs between constipation and controls

**Figure 4:**
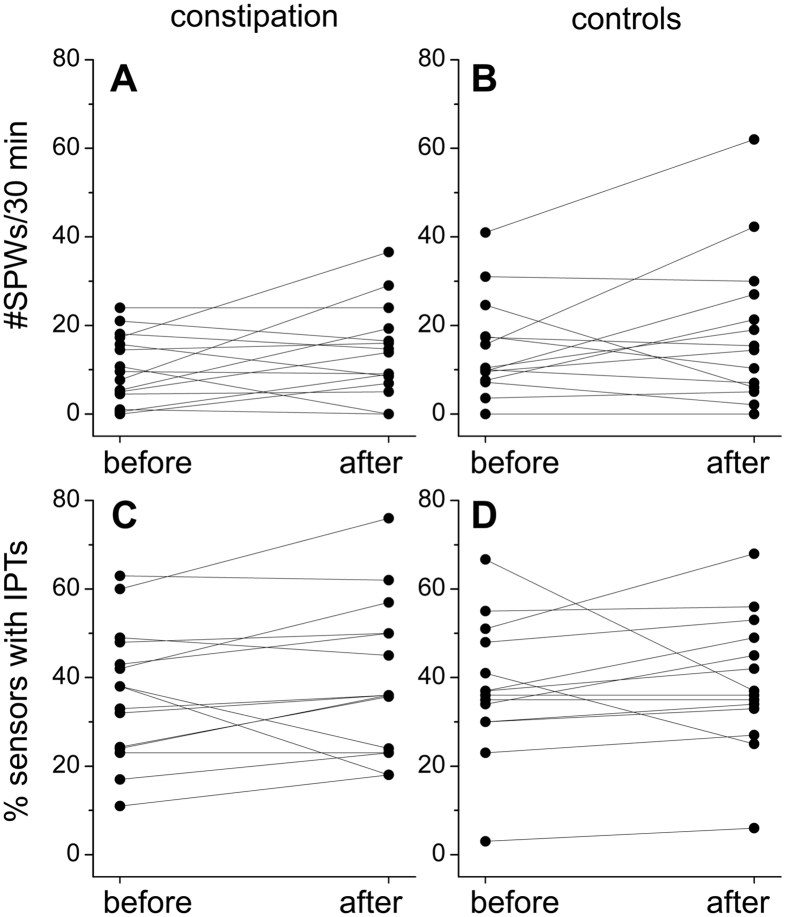
Individual changes in the occurrence of SPWs and isolated pressure transients in response to a meal. (**A,B**) Expressed are the average number of SPWs occurring in a 30 min period before and after the meal in each subject from the patients with chronic constipation (A; n = 15) and the combined controls (B; n = 14). (**C,D**) Expressed are the % sensors that displayed isolated pressure transients before and after the meal in each patient with chronic constipation (C; n = 15) and the combined controls (D; n = 14).

Comparison of parameters within and between patient controls and volunteer controls is shown in supplementary Table S1. Within the two groups, based on the average values, no differences in the occurrence of motor patterns before or after a meal were observed and almost all parameters were not different; only in patient controls the SPW duration decreased after a meal, whereas in the volunteer controls, the SPW amplitude decreased after a meal. Comparing the two groups, only the amplitude of the SPWs after a meal was found to be significantly greater in the patient controls. Comparison between patients and all controls (Table 1) indicates that most patients who relied on laxatives for bowel movement, showed the ability to generate normal motor activity. Statistical difference was found in the maximum SPW frequency, which increased after the meal in the control group, whereas the propagation distance decreased in the constipation patient group. To get insight into activities in each subject, the occurrence of SPWs before and after the meal are given in Fig. 4. In the constipation group, 7 out of 15 patients showed a numerical increase in the number of SPWs in response to a meal, in the combined control group, this was 7 out of 14.

All patients in the constipation group relied on laxatives for bowel movement and all were diagnosed (prior to HRCM and in other centers) with an inert colon based on symptoms and transit studies. Based on HRCM, all patients showed varying degrees of colonic motor activity and all were advised to try to wean off laxatives, during which time they took prucalopride 2 mg p.o. for 3 months and were advised on optimizing their diet and exercise. 8 patients responded such that no laxatives were used after 1 year, 3 patients continued to rely on laxatives and 4 patients were not available for follow up. Supplementary Table S2 compares the data between the responders (no laxatives after 1 year) and the non-responders (continued to rely on laxatives after 1 year). The number of SPWs in the responder group was 12.5 SPWs /30 min (5.0–21.0) (n = 8) at baseline, and 18.5 (8.5–36.6) SPWs/30 min after the meal. The non-responders had 5.3 SPWs/30 min (0.5–14.5) at baseline and 8.3 SPWs /30 min (0–16.0) (n = 3) after the meal. Statistical analysis was not done because of n < 5.

### Isolated pressure transients

Isolated pressure transients were defined as short-lasting pressure increases, ≥ 5 mmHg, present in one sensor and not connected to other pressure changes in adjacent sensors. Considering all subjects, the number of sensors exhibiting isolated pressure transients ranged from 17–68%. When isolated pressure transients were present, almost all sensors exhibited pressures of < 50 mmHg; sensors that exhibited pressures between 50–100 mmHg ranged from 0 to 81%; sensors exhibiting pressure transient > 100 mmHg ranged from 0–40%. The maximum amplitude of the pressures achieved in a particular 30-min period ranged from 21 to 223 mmHg.

Comparing patients with the combined control subjects, the average values of the features of the isolated pressure transients before or after a meal in both groups and between controls and constipation were not significantly different (Table 1) except that in controls the percentage with amplitudes between 50–100 mmHg decreased after the meal.

**Figure 5:**
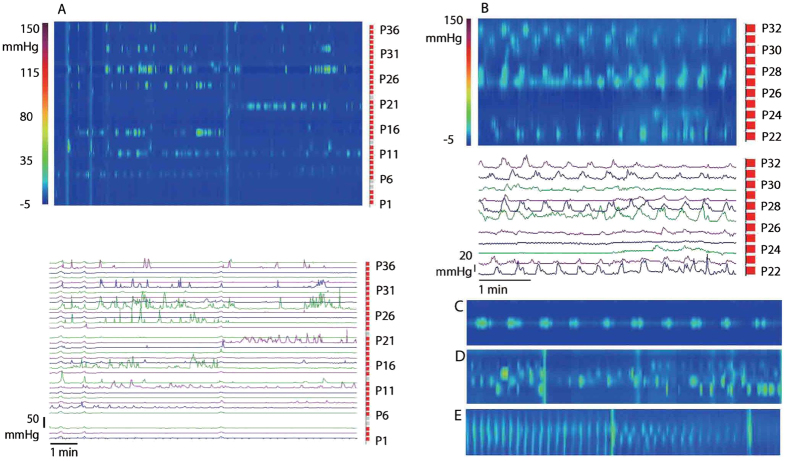
Haustral Activities. (**A**) Isolated pressure transients that are confined to regularly spaced sensors. Here 8 sensors are showing isolated pressure transients, the sensors are 4 cm apart, likely reflecting narrowing of the lumen created by haustral boundary contractions. Of note is that this pattern does not appear to propagate to other sensors. Control subject, baseline. Sensor P36 is 45 cm from anal verge. (**B**) Rhythmic 3 cycles/min pressure transients dominate the activity. The activity centers on sensor 31, 27 and 22, which is in the order of the spacing of haustral boundaries. From a patient with constipation; sensor P36 is 75 cm from anal verge. (**C**) Isolated pressure transients occurring at a sustained 3 cycles/min rhythm, no activity is seen in adjacent sensors. Duration of recording is 3.5 min. This represents 4 cm of colon covering 5 sensors. (**D**) Activity in 5 consecutive sensors occurring in an apparent erratic manner, and with marked differences in pressure amplitude. This activity is very similar to that occurring within haustra of the rabbit colon^25^ and similar to segmentation activity in the small intestine^17^ hence identified here as segmentation. The duration of the recording is 17 min. The figure covers 5 cm of the colon. From a patient with constipation. (**E**) From the same patient as (**D**). The erratic activity changed into synchronized activity; at the half point of the recording, the synchronization decreases. The duration of the recording is 17 min. The figure covers 5 cm of the colon. This figure is a small part of Fig. 6A.

Considering individual subjects, the change in the percentage of sensors displaying isolated pressure transients in response to the meal is given in Fig. 5. In the constipation group, 10 out of 15 patients showed a numerical increase in the number of sensors exhibiting isolated pressure transients in response to a meal, in the combined control group, this was 11 out of 14.

### Haustral boundary pressure transients

A very common pressure pattern was a continuing display of isolated pressure transients at sensors spaced 3–4 cm apart, with little activity at the sensors in between (Fig. 5A,C). When present, the pressure pattern remained associated with the same sensors for 30–180 min. The rhythmicity could be irregular but it was often of a strikingly constant frequency of ~3 cycles/min (Fig. 5B,C). When SPWs occurred, they often had pressure transients superimposed with the strongest ones separated by 3–4 cm (Fig. 1), suggesting that when the pressure wave moved across these sensors, the lumen was narrowest there and the pressure highest. This activity appears to reflect haustral boundary activity.

### Intrahaustral segmentation activity

When isolated pressure transients were seen in 2–5 adjacent sensors, a complex pattern could emerge (Fig. 5D), similar to the segmentation pattern in the small intestine^17^. The pattern was most often seen in not more than 5 consecutive sensors, it appears to be activity within a haustrum. The average time this intrahaustral segmentation pressure activity was seen in all subjects, was not significantly influenced by a meal nor was it significantly different between constipation and controls.

### Synchronized Haustral Pressure Waves

The intrahaustral segmentation activity alternated with the activity being synchronized over the 4–5 sensors, propagating at 3 cycles/min, at 2 ± 1 cm/s in antegrade or retrograde direction, becoming “synchronized haustral pressure waves” (Fig. 5E). These waves occurred for 2 to 10 min, 1–3 times during a session or not at all (Table 1). The switch from segmentation to synchronization or from synchronization to segmentation was seen in all parts of the colon where it was recorded.

Synchronous haustral pressure waves alternating with intrahaustral segmentation activity were evoked by or increased after the meal in 9 patients with constipation (Table 1; P = 0.008). In controls, they appeared after a meal in 4 subjects. This suggests that haustral activity is more prominent in patients with constipation compared to controls and that it is induced by a meal.

**Figure 6:**
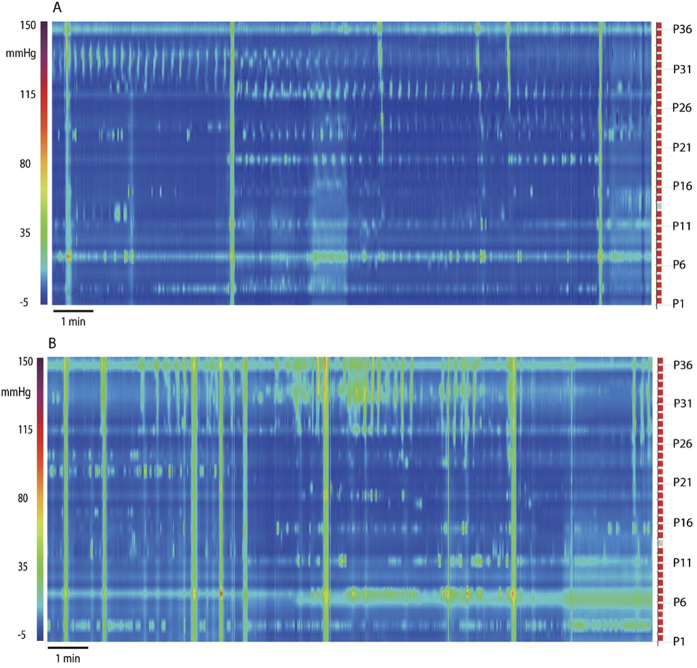
Response to a meal involving simultaneous pressure waves as well as segmentation and synchronized haustral pressure waves. (**A**) A typical example of synchronous haustral pressure waves, showing in sensors 28–34 in the first 4 min of the baseline recording from a patient with constipation. After 4 min, an SPW occurs. The synchronized activity is only seen in 4–5 sensors making it likely that it involves activity in a single haustrum. The activity is stably rhythmic at ~3 cycles/min. (**B**) The same patient as shown in (**A**), activity after the meal. SPW activity increases as well as the synchronous haustral pressure waves. Isolated pressure transients also increase.

To further illustrate the occurrence of synchronized haustral pressure waves, Fig. 6 shows the effect of the meal in a 52-year old female who depended on daily Senna laxatives for bowel movements for 3 years. A shapes study showed 75% (15 out of 20) radiopaque markers remained in distal colon after 72 hours; she had one bowel movement every 4–5 days prior to starting the use of laxatives. The patient came to the clinic because of worries about chronic laxative use. All motor patterns were present except HAPWs and Retrograde Propagating Pressure Waves (RPWs) at baseline. In the 30 min prior to the meal, synchronized haustral pressure waves were present in the proximal part of the colon segment studied; they markedly increased in amplitude after the meal. The activity showed strong rhythmicity at 2.6 cycles/min. The meal also evoked RPWs. Isolated pressure transient amplitudes were between 10 and 60 mm Hg.

### High Amplitude Propagating Pressure Waves (HAPWs)

**Figure 7:**
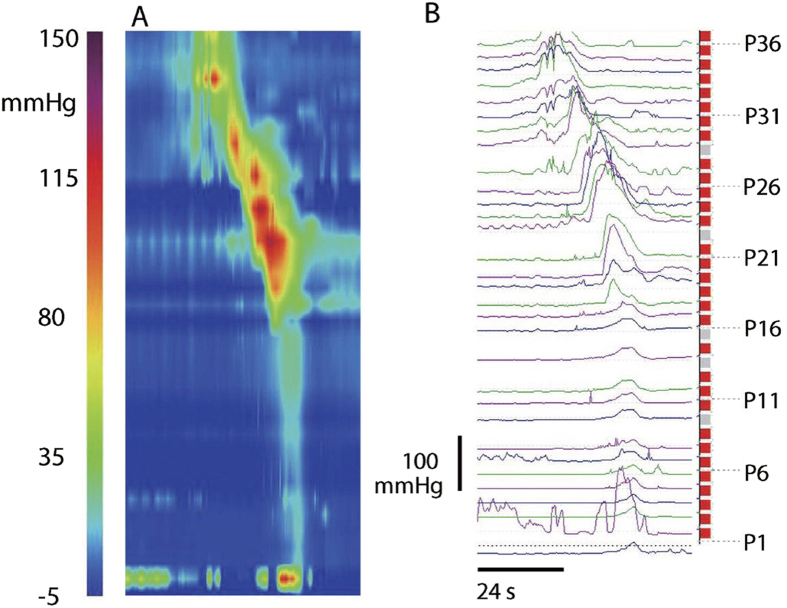
High Amplitude Propagating Pressure Wave (HAPW). An HAPW in a spatio-temporal pressure map (**A**) and seen as pressure traces (**B**) Control subject, observed in response to the meal. Sensor P36 was 70 cm from anal verge.

HAPWs were observed after a meal in 2/14 control subjects (Fig. 7), and in 2/15 patients with chronic constipation (Table 1).

### Antegrade propagating pressure waves (APWs) and retrograde propagating pressure waves (RPWs)

**Figure 8:**
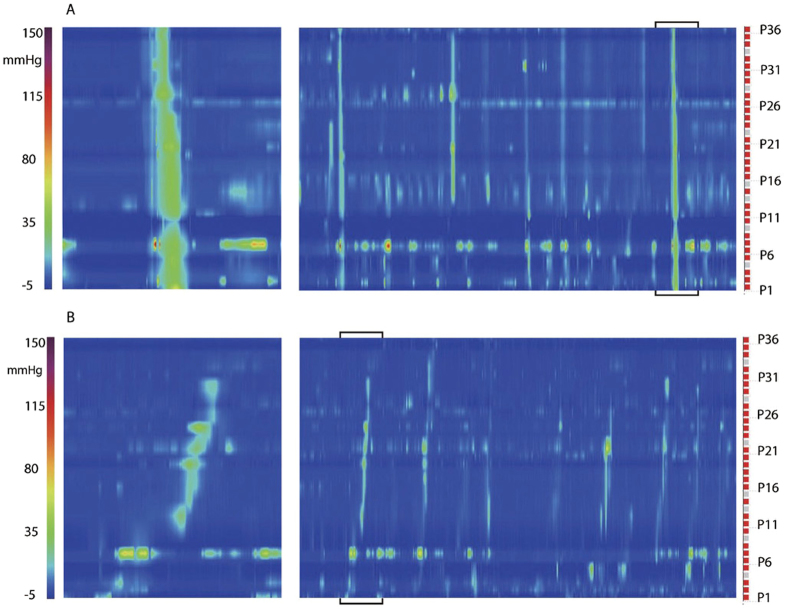
Antegrade (APWs) and retrograde (RPWs) propagating pressure waves. (**A**) An APW in a one min section, which is an enlargement from the bracketed section in the 10 min recording. Recording is from a control subject. Sensor P36 is 70 cm from anal verge. (**B**) RPWs from the same individual but recorded after the meal.

APWs were identified as antegrade propagating waves when the amplitude was ≥5 mmHg but <100 mmHg observed over three or more adjacent sensors. Considering all subjects, the propagation velocity of APWs was in the range of 4.8–7.2 cm/s, the propagation velocity of RPWs was in the range of 0.6–3.7 cm/s (Fig. 8). APWs occurred either as isolated events or in a rhythmic manner in the range of 0.5 to 3 per min. The average amplitude across all 36 sensors of APWs occurred in the range of 11 to 53 mmHg but the amplitude was never consistent over all 36 sensors. APWs often had associated isolated pressure transients.

Comparing patients with constipation and combined controls (Table 1), there were no significant differences in response to a meal and between constipation and controls (Table 1).

APWs were observed in 4 out of 15 constipated patients at baseline and 6 out of 15 showed APWs when both baseline and the meal were considered. In these six patients, the average value of APWs was 1.0 ± 1.0 /30 min before, and 3.7 ± 4.0 /30 min after the meal. In controls, APWs were recorded in 4/13 at baseline and 7/13 after the meal, the average values were 1.7 ± 2.3 before and 3.4 ± 4.6 APWs/30 min after the meal.

RPWs were observed in 10/29 subjects. Considering all subjects, there were no differences in RPW parameters before or after a meal. Comparing patients with constipation and combined controls (Table 1), retrograde pressure waves (RPWs) were seen in 1/15 constipated patients at baseline and 4/15 showed RPWs when both baseline and the meal were considered. The occurrence ranged from 1/30 min to 14/30 min. In controls, RPWs were recorded in 2/14 at baseline and 6/14 after a meal with the occurrence ranging from 1/30 min to 11/30 min.

## Discussion

The objective of a colon motor function test is to investigate the capability of a patient to generate “spontaneous” and stimulus-induced colonic motor activity. In most studies comparing a cohort of patients with a cohort of controls, statements are made whether or not, on average, a particular motor pattern is increased or decreased, although mostly only HAPWs are evaluated and sometimes a statement is made whether or not overall activity was increased after a meal. The present HRCM study identifies many more motor patterns and identifies them as propagating or non-propagating, promoting transit or absorption, and so gives a better assessment of motor function. The present study also shows that most patients with chronic constipation who rely on laxatives for bowel movements, and who have been diagnosed having an inert colon 3–25 years prior to the study, show marked contractile activity and responses to a meal. For these patients, this was a revelation that encouraged adherence to treatment; the treatment of short-term prokinetics in addition to life style changes resulted in 8 out of the 11 patients that were available for follow up obtaining regular bowel movements without laxatives within 1 year. Our data suggest the hypothesis that patients that rely on laxatives for bowel movements, who show a high baseline level of SPWs (10–20/30 min) and/or a significant increase of SPWs (200–300%) and an increase in haustral activity in response to a meal, will respond to short term prokinetics and life style changes so that laxatives may no longer be needed after 3 months to 1 year.

The present study shows that motor patterns and responses to a meal vary markedly in controls and in patients with constipation who rely on laxatives for bowel movements. No unique motor pattern associated with constipation was observed but patients with constipation evoke haustral activity in response to a meal more compared to controls suggesting stronger absorption. A definitive search for statistical differences in motor patterns between controls and patients with constipation requires larger sample sizes as well as a search for optimal stimuli to evoke distinct motor patterns. What follows is a discussion on the distinct pressure patterns that were identified.

SPWs were observed as transient increases in intraluminal pressure, most often covering the entire 35 cm of the colon assessed. The term simultaneous pressure wave was chosen as it was defined by De Schryver *et al*. and Rao *et al*.^18^,^9^. This motor pattern has also been referred to as simultaneous contractions^16^,^19^ or simultaneous waves^20^. The term “pressure” was chosen over “contraction” since manometry measures pressure only.

In the present study, SPWs were by far the most visually prominent propulsive motor pattern. The propulsive character of SPWs is related to their prominent association with gas expulsion as determined in the present study and by Kamm *et al*.^21^. Furthermore, currently we perform HRCM with 84 channel water-perfused catheters, and SPWs of amplitudes above 40 mmHg cause outflow of water in healthy volunteers (Chen and Huizinga, unpublished). The SPWs were associated with relaxation of the internal anal sphincter, even if the SPWs were of low amplitude. The latter puts the SPWs in the same category as other propulsive motor patterns such as the HAPW^22^ and esophageal and gastric propagating motor activities that are followed by sphincter relaxation^23^,^24^.

Simultaneous pressure waves were not caused by a contraction in any part of the measured segment that simply caused pressure changes in rest of the segment by what is often called a “common cavity” effect. This is best illustrated by the fact that an HAPW that occurred in the proximal part of the measured segment did not immediately cause a simultaneous pressure change in the distal part. A high amplitude pressure wave of 120 mmHg, traveling over 10 cm in the proximal part of the measured segment, may not cause any change in pressure in the distal part of the measured segment. In unpublished studies we simultaneously measured motor patterns and intraluminal pressure by HRCM in the proximal colon of the rabbit and showed that a simultaneous pressure wave is the consequence of fast propagating, propulsive contractions. The general nature of motor patterns in the rabbit has been described previously^25^,^26^,^27^,^28^,^29^. Furthermore, numerous observations reported in the literature indicate the prominence of fast propulsive movement of content in the human colon as seen with barium X-rays. Ritchie noted that “content moved almost instantaneously from the hepatic flexure to the splenic flexure”^30^. Hertz and Newton noted that “not more than a second or two was occupied in the passage of the faeces from one end of the colon to the other”^31^. Fast propagating contractions are most likely related to fast “rushes” of electrical activity recorded from the human colon by Bueno *et al*. propagating over ~100 cm at a velocity of 10.5 ± 2.6 cm/s^32^. Waves of such velocity will appear simultaneous in the present study and in 24 hr studies such as those done by Rao and coworkers^9^. De Schryver *et al*. noted that pressure waves could propagate with velocities up to 12 cm/s^18^. Interestingly, Bueno *et al*. found that the electrical “rushes” were more prominent in patients with constipation compared to those with diarrhea^33^.

Although simultaneous pressure waves were “the predominant pattern of motor activity in the colon” shown in a 2001 study using 24 hr manometry^9^, in most colonic studies to date, they are not evaluated, likely because of fear to include artifacts due to abdominal pressure changes. In the first 8 hours of a 24 h recording, the number of “simultaneous contractions” was not different between controls and constipated patients^10^. De Schryver *et al*. noted that the incidence of simultaneous pressure waves was significantly higher in patients with constipation compared to the healthy subjects (18 ± 5 vs 6 ± 3 per 2 hrs), but no effect of a meal was observed^18^. In these studies from Rao *et al*. and Schryver *et al*., no mention was made of responses in individual patients. In one study in children, simultaneous pressure waves were seen only after bisacodyl^16^ and primarily in the distal colon and then often as a continuation of HAPWs. In the present study, SPWs were common in controls and patients with constipation and many patients with constipation and controls showed a numerical increase in SPWs after the meal.

The fast propagating contractions underlying the SPWs, as characterized in the rabbit colon, are fundamentally a myogenic motor pattern since they can occur in the presence of TTX^25^,^27^ or in the presence of hexamethonium^27^. SPWs are often highly rhythmic at ~0.5–1 cycle/min suggesting that a pacemaker is associated with the motor pattern. Interstitial cells of Cajal (ICC) are pacemakers of gut motility^27^,^34^,^35^,^36^. The fast contractions in the rabbit colon occur synchronously in the circular and longitudinal muscle^27^ suggesting the likelihood that ICC associated with the myenteric plexus (ICC-MP) are the associated pacemaker^25^,^27^. Reported intracellular electrical and mechanical activities of the human colon *in vitro* suggest that in the human colon, low frequency (0.3–0.6 cpm) slow waves are associated with the ICC-MP^37^. A 1–3 cycles/min electrical rhythmicity was also observed in the human colon circular muscle using extracellular electrodes^38^. The pacemaker activity of the ICC-MP in the colon is stimulus dependent^37^,^39^,^40^ with distention and neural activity as possible stimuli. Fast propagating contractions become more effective as a propulsive motor pattern when they occur in clusters of 2 or more contractions; under these conditions, the activity is inhibited by TTX^25^ and hence neurally regulated. An SPW will be associated with a cluster of fast propagating contractions. A neurogenic component is also suggested by the close relationship between an SPW and relaxation of the internal anal sphincter.

Isolated pressure transients, pressure increases recorded by one sensor and not connected to a pressure change in adjacent sensors, were seen in every subject under all conditions. They are also referred to as Isolated Pressure Waves^18^,^16^. Isolated pressure transients often appear in a very regular pattern: on every 3^rd^ or 4^th^, occasionally every 8^th^ sensor, for long periods of time (Fig. 5). Importantly, the activity is often highly rhythmic at a remarkably constant frequency at ~3 cycles/min. With the definition of a haustrum^41^ being a sac-like structure that is separated by two active circumferential circular muscle contractions about 3–4 cm apart, the transient pressure increases are likely haustral boundary activity hence we identified this motor pattern as rhythmic haustral boundary pressure transients. This is consistent with the highly rhythmic haustral boundary contractions in the rabbit proximal 3-taeniated colon^25^,^26^,^27^. In the rabbit, haustral boundary activity slowly propagates and is neurogenic since it is sensitive to hexamethonium^27^ or tetrodotoxin^25^. In the rabbit, the haustral boundary contractions are rhythmic at 8 cycles/min associated with interstitial cells of Cajal associated with the submuscular plexus (ICC-SMP)^25^,^27^. In the human colon, the 3 cycles/min rhythmicity of the haustral boundary pressure transients is most likely also associated with electrical slow wave activity generated by ICC-SMP occurring in the range of 2–4/min as was observed in human tissue *in vitro* using intracellular electrodes^42^.

Synchronized haustral pressure waves relate to our observation that the 3 cycles/min contractions, so often seen as haustral boundary contractions, can propagate over 4–5 adjacent sensors (Figs 5E, 6) hence apparently restricted to a single haustrum. This activity is likely the same as what has been referred to as periodic colonic motor activity^10^ or cyclic propagating motor pattern^43^ or discreet random bursts of phasic pressure waves^16^. Other studies also show the pattern to be extended over the length of two or more haustra^43^. This pattern may be related to Retrograde Propagating Sequences^44^ but we did not see them consistently occurring retrograde. In other studies, the 3 cycles/min rhythmicity was seen as the dominant frequency of phasic contractions after a meal^45^ and the dominant frequency of cyclic motor patterns^46^. In 24 hour recordings, Rao *et al*. noted a predominance of bursts of pressure waves at 3 cycles/min^9^. In a recent HRCM study in children, discrete random bursts of phasic pressure waves occurred at 3 cycles/min^16^.

The synchronized haustral pressure waves occur at the frequency of the dominant pacemaker, the ICC-SMP. Slow waves originating in ICC-SMP have been recorded in the human colon *in vitro*^42^,^47^ and *in vivo*^48^. *In vitro*, this activity was not sensitive to nerve blockade, confirming that a myogenic pacemaker is involved^47^,^49^. This suggests them to be equal to the ripples identified in the rabbit colon^25^,^26^,^27^.

In a study comparing constipation to controls, Dinning *et al*. described the synchronized haustral pressure waves as cyclic motor activity at a frequency of 2–6 cycles/min, occurring retrograde or antegrade over a distance of 4.4 to 8.2 cm; it was noted, based on group comparison, that the normal increase in this activity after a meal was absent in patients with chronic constipation; although in 5 patients where no activity was seen preprandial, it was observed post prandial (Table 1 in^43^). De Schryver saw short antegrade propagating activity increase after a meal in controls but not in constipation^18^. In the present study, within clusters of synchronous haustral pressure waves, the waves were almost always of mixed direction or appearing simultaneous. The synchronized haustral pressure waves showed a significant increase in patients with constipation after a meal (Table 1); always alternating with un-synchronized, apparent erratic activity (Fig. 5D), resembling the segmentation activity of the small intestine^17^.

High amplitude propagating pressure waves (HAPWs) were the most forceful propulsive motor pattern. Since HRCM measures pressure and not contraction we used the term HAPW, similar to a study by De Schryver *et al*.^18^, although this motor pattern is often called High Amplitude Propulsive Contraction (HAPC), e.g.^50^,^51^. It is also called High Amplitude Pressure Sequence^16^,^46^ or Specialized Propagating Pressure Waves^9^. In a comparative 24 hr study of chronic constipation and healthy volunteers, 2.5 HAPWs were seen per 6 hours on average, including meals and awakening and HAPWs were observed less frequently in chronic constipation; it was suggested to be a useful marker for constipation^9^,^10^. However, even 24 hours recording may not show the motor pattern, even in healthy volunteers^52^. In a 4 hr test including a meal, only 5 of 12 controls showed HAPWs in response to the meal, and only 1 out of 14 patients with chronic constipation^43^. Another study showed HAPWs in response to a meal in 1 out of 10 patient with chronic constipation and in 4 of 10 healthy individuals^18^. In the present study, HAPWs were observed after a meal in 2/14 control subjects and in 2/15 patients with chronic constipation. In children, the absence of HAPWs in response to bisacodyl is considered abnormal and reason to consider surgery^52^; we do not consider the absence of HAPWs in chronic constipation in adults in response to a meal to identify an inert colon, since many other significant motor patterns may exist. HAPWs can be followed by a short period of relative quiescence but we did not see a relationship between quiescence and constipation as shown to occur in children^16^. Since HAPWs can be evoked by a meal (due to a gastro-colonic reflex) or by bisacodyl^16^ it is likely a motor pattern generated by a neural program that is initiated by a stimulus. A low or high calorie meal^43^ is not sufficient to identify if a patient has the capability of generating this motor pattern but it does give an indication of the sensitivity of the nervous systems to a meal. It is likely that a variety of stimuli, including bisacodyl, will best assess if the patient is capable of generating a HAPW.

In the rabbit colon, when measuring diameter changes, hence circular muscle contraction, this most forceful type of peristaltic activity has been referred to as mass peristalsis^27^ or long distance contraction^25^ or colonic migrating motor complexes^26^. In mouse models, it is referred to as colonic migrating motor complexes (CMMCs)^39^,^53^,^54^, in the rat colon as long distance contractions (LDCs)^40^,^55^. In all the animal models studied, this motor pattern is neurogenic, hence the HAPW in the human colon can be considered neurogenic, consistent with the observation that it is induced by awakening or a meal^56^; consequently, the absence of HAPWs has been suggested to reflect a neuropathy^57^.

Propagating pressure waves that are of lower amplitude than HAPWs, are most likely very significant for transit. As pointed out by Dinning *et al*. even if they do not cover a large section of the colon, sequential APWs should be effective in transit^58^. Hence they should be part of the normal assessment of motor function, also because they can signify a response to a stimulus such as a meal. We used the term “pressure waves” as was done in studies by Rao *et al*.^9^,^20^ and De Schryver *et al*.^18^. Other terms used are Propagating Sequences (PS)^14^, Propagating Contractions^59^ or Retrograde Waves^20^. Using fiber-optics high-resolution manometry, Dinning *et al*. referred to these as long or short single motor patterns and no differences were found between controls and patients with constipation^43^. An example for comparison: in Dinning’s study, the antegrade single short and long propagating motor pattern in constipation occurred 2.6 times/2 h before and 3.0 times/2 h after a meal; in our study, in patients with constipation, APWs occurred in 6 out of 15 patients, in which they occurred 5.6 before and 14.8 times/2 h after the meal. In both studies, the difference was not significant because of high variability. In Rao’s study, antegrade propagating pressure waves in controls occurred 5 times/2 h on the first day of recording vs 3.5 times/2 h in patients with constipation; the difference was not significant^10^. With respect to retrograde activity, in Rao’s study, retrograde activity was not often found and if it did, it occurred after a meal but there was no difference between controls and constipation^10^. In Dinning’s 6-hour study, no differences in retrograde activity were found before and after a meal^43^. In our study, also no difference was found.

In the present study, the occurrence of APWs after a meal showed the capability of the colon to generate propulsive activity. In subjects with significant APW activity, no urge of bowel movements was noted; this is likely because APW activity did not proceed into the rectum. The occurrence of RPWs was also a sign that neurally generated activity can be performed.

The electrical activity that is causing propagating contractions and the consequent propagating pressure waves is likely a slow wave with superimposed trains of action potentials, recorded using luminal electrodes as long spike bursts (LSBs)^32^. The duration of most propagating contractions in all subjects was between 10 and 30 s, which is similar to the average duration of long spike bursts (LSBs, 19.6 ± 2.5 s). The LSBs propagated at 4.2 cm/s and the APWs in the present study between 2 and 5 cm/s. The LSB frequency was 23 per hour but often occurring in bursts at 2–3 /min, similar to the APWs.

Surgical pathology has revealed that a common feature of patients with chronic constipation is a decrease in the number of ICC pacemaker cells^60^,^61^,^62^,^63^. The different ICC networks are not always differentiated in surgical pathology, making it difficult to predict which motor patterns may be affected by the loss of ICC. At this moment we do not know how much loss of cells the ICC network can absorb before loss or impairment of function becomes evident^64^,^65^. Lammers *et al*. suggested that as much as 35% loss may be required to affect ICC-MP slow wave activity in the small intestine^66^. More research is needed to substantiate this for all networks and in the colon. Since ICC are also involved in neurotransmission^67^,^68^,^69^, more subtle changes in the ability to develop adequate motor patterns may be due to loss of ICC-mediated innervation.

One of the limitations of the present study is that it covered a limited section of the colon, 35 cm, focusing on the transverse – descending colon; since colonic dysfunction may be limited to the proximal colon or sigmoid colon, assessment of pan-colonic motility is ideal. Another limitation is that the yoghurt meal, a meal considered a full meal by all participants, was a relatively low calorie meal which may have had a low sensitivity for the induction of HAPWs and other motor patterns compared to a higher calorie meal, although HAPW findings similar to the present study were encountered with a higher calorie meal by Dinning *et al*.^43^.

While the present study was under review, Corsetti *et al*.^70^ also identified the SPWs as an important motor pattern in the human colon and confirmed its association with internal anal sphincter relaxation and gas expulsion^71^. They showed that patients with chronic constipation refractory to current pharmacological treatments presented a decrease in the occurrence of SPWs^70^.

Much more information about motor dysfunction is needed before certain motor patterns can define patient populations. The current value of HRCM lies in its ability to reveal normal pressure patterns or motor dysfunction in each patient and this knowledge can help individual management. The challenge is to identify a set of stimuli in motility testing that will give full understanding of the capabilities of the colon. Likely, no single stimulus will suffice. In children, bisacodyl stimulation is used to evaluate the capability of generating HAPWs but this is not commonly performed in adults, although its use in adults will likely increase. In children with constipation, intraluminal bisacodyl often works to create HAPWs even if bisacodyl is not effective as a treatment^16^. This may suggest that in the patients’ daily life, the sensitivity to stimuli is reduced. Herve *et al*. showed that spontaneous HAPWs were significantly lower in number in patients with constipation versus controls but there was no difference with bisacodyl-induced HAPWs^59^. It is our goal to identify stimuli that evoke propulsive or non-propulsive activities and understand their mechanism of action so that failure to respond to the stimulus *or a normal response to the stimulus*, gives information about the state of colon function and mechanisms underlying dysfunction. In addition, it needs to be studied what underlies low sensitivity in patients; possible causes are eating habits, exercise routines, stress, microbiota composition. It may also relate to abnormalities in intrinsic and extrinsic innervation or reduction in ICC.

## Methods

High Resolution Colonic Manometry (HRCM) was performed on the transverse – descending colon, using a 36-sensor solid-state manometry probe (Unisensor Attikon, Switzerland) with 1 cm distance between sensors. Hardware and acquisition software were provided by MedKinetic Inc (Ningbo China). The study was conducted at Renmin Hospital at Wuhan University. The study was approved by the “Medical Ethics Review Committee of Renmin Hospital, Wuhan University”, related to the National Natural Science Foundation of China (NSFC) funded project No. 81170349. Informed consent was obtained from all participants. All methods were performed in accordance with the guidelines and regulations of the National Natural Science Foundation of China and the Medical Ethics Review Committee of Renmin hospital. The catheter was inserted without sedation with the help of a colonoscope after a routine cleaning procedure using polyethylene glycol electrolytes powder (Fortrans, Beaufort Ipsen Industrie, France). The tip of the catheter was clipped to the mucosa at about the middle of the transverse colon via a cotton thread. The position of the tip of catheter was noted as the length in cm inside the body relative to the anal verge, and noted in all figures. The most proximal sensor of the catheter is identified as P36 as shown in all figures. In patients with constipation, the catheter tip was 62.5 ± 1.8 cm from the anal verge (n = 15), whereas in controls this was 59.6 ± 1.7 cm (n = 14), not significantly different. In 3 patients, after the full procedure was performed, the catheter was re-positioned such that the internal anal sphincter pressure could be recorded.

Patients and volunteers were recruited in the first 6 months of 2013 from the motility clinic at Renmin hospital that specializes in constipation and gastroesophageal reflux. Exclusion criteria were: abdominal surgery, hepatic, kidney or cardiac diseases, connective tissue disorders, CNS disorders, thyroid diseases, prostate diseases or cancer. Medications or laxatives affecting bowel function were withheld for 3 days prior to the study day.

Two control groups were studied. One group consisted of 5 healthy volunteers who did not have any bowel complaints, and they were not on any medication that could be expected to have an effect on bowel function. Another group consisted of 9 patients from the motility clinic. Similar to all such clinics in China, this is a walk-in clinic where anyone can go without referral and hence without pre-screening. About 90% of patients visiting the clinic are new and many have vague complaints and their main goal is to get screened for gut abnormalities including colon cancer. The 9 patients chosen to be included in the present study had mild anxiety and IBS-like symptoms, including short-term concerns about stool form or stool frequency, but without pain and without any sign of constipation. These 9 patients could not be classified as IBS according to Rome III criteria; follow up after one year confirmed this, there were no symptoms at that time; they volunteered to do colonoscopy and the HRCM study, which they saw as a method to rule out colonic abnormalities including cancer.

All subjects were in the supine position during the entire recording, except during intake of the meal. The subjects were asked to tell us all events such as gas expulsion, bowel movements, pain and discomfort. All events were noted immediately into the data acquisition files by one of the authors. All body movements such as changing body position, talking and coughing were noted immediately into the data acquisition files.

Spatiotemporal pressure maps show color-coded bars that are indicating the intraluminal pressure. The red squares at the right side of the maps indicate the measurement sensors. A gray square indicates a malfunctioning sensor.

The analysis was done using the acquisition and replay software developed by MedKinetic^®^, as well as Image J^®^ and Excel^®^. There is no analysis software to automatically analyze colon motor patterns reported here. Preliminary data were published in abstract form^71^,^72^,^73^,^74^.

### HRCM procedure

Following the placement of the catheter, baseline activity was recorded for 90 min. Thereafter, all subjects received 460 g yoghurt “Bright e+ probiotic fermented milk^®^” with the following specifics: energy 1329 kJ, protein 12.4 g, fat 13.3 g, carbohydrates 36.8 g, sodium 276 mg. Whole raw milk with probiotics: Lactobacillus bulgaricus, Streptococcus thermophiles, actobacillus acidophilus, Bifidobacterium bifidum, Lactobacillus casei. Additives: white sugar, pectin, aspartame, phenylalanine, acesulfame.

This meal was chosen because it was attractive to the subjects; it gave the opportunity to discuss healthy food choices and the meal was considered by all participants to be a full meal.

The response of the meal was evaluated for 90–120 min. Patients were kept awake during the study and followed the procedures on a monitor. Talking, coughing, body movements, gas and discomfort were recorded.

### Treatment

After the HRCM procedure, all patients were interested in trying to wean off laxatives. All patients took prucalopride for 3 months and were advised to increase exercise and eat healthy. A telephone interview at 3 months and 12 months after HRCM by Dr Chen was conducted in 11 of the 15 patients. 4 patients were not available for follow up.

### Simultaneous pressure waves (SPWs)

A simultaneous pressure wave (SPW) was identified as a transient pressure increase of at least 2.5 s duration, with an amplitude of at least 5 mmHg that appeared to occur simultaneously in most or all sensors, if not an artifact (see results). The following parameters were analyzed.

#### Propagation velocity

Since propagation velocity could be determined accurately only up to ~7 cm/s, the propagation velocity was at least 7 cm/s and this pressure pattern was described as occurring simultaneously.

#### Occurrence

SPWs were identified in the baseline period and after a meal. These periods lasted 90–120 min and the # of SPWs was expressed as average number per 30 min.

#### Maximum frequency

If a period of regular rhythmic activity occurred, its frequency was determined. Often, irregular activity alternated with a period of a sustained rhythmicity, the maximum frequency was the frequency within the period of sustained rhythmicity. The maximum frequency was measured to obtain an indication of the pacemaker frequency underlying the activity. Unlike the heart, ICC pacemaker activity does not inevitably lead to contraction. Although the musculature receives the rhythmic depolarization; it may not be enough to pass threshold for contraction (calcium channel activation) hence another stimulus may be required to make this happen. A period of highly rhythmic activity with a constant frequency may indicate the maximal contraction frequency, hence the pacemaker frequency.

#### Average pressure duration

The duration of the pressure increase was measured as the time between initial rise and end of the amplitude increase.

#### Average pressure distance

The distance between the first and last pressure sensor was 35 cm. The length of the simultaneous pressure wave was expressed in cm.

#### Average amplitude

The cursor placed at the center of the SPW gave the amplitude at all 36 points and the average was calculated and expressed as mmHg.

### Propagating pressure waves

Propagating pressure waves were identified if a propagation velocity could be determined. They were at least 5 mmHg in amplitude but less than 100 mmHg. They were propagating antegrade (APW) or retrograde (RPW).

If the amplitude of an antegrade pressure wave (APW) was equal to or higher than 100 mmHg in at least 3 sensors, the pattern was classified as a high amplitude pressure wave (HAPW).

The parameters analyzed were the same as those for SPWs.

### High Amplitude Pressure Waves

If the amplitude of a propagating pressure wave was >100 mmHg in 3 or more sensors, the wave was classified as a high amplitude pressure wave. It was determined whether or not the pattern occurred before and/or after a meal.

### Isolated pressure transients

Isolated pressure transients were defined as short-lasting pressure increases, present in one sensor and not connected to other pressure changes in adjacent sensors. Their amplitude ranged from 5 mmHg to > 100 mmHg.

#### Maximum amplitude

The maximum amplitude of the isolated pressure transients observed in each sensor was determined and the average was reported as mmHg.

#### Percentage sensors involved

All sensors were scanned by eye for the presence of isolated pressure transients and the number of sensors involved, was divided by 36 to calculate the percentage of sensors that recorded this pattern.

#### Percentage time involved

For each sensor, the percentage of time the activity occurred was calculated and the average of all sensors was reported.

*Percentage with amplitude* <*50* *mmHg; percentage with amplitude 50*–*100* *mmHg; percentage with amplitude* >*100* *mmHg:* From the sensors that showed activity it was determined whether or not they exhibited activity in these three categories. Since many sensors recorded pressures in more than one category, the total percentage of the activities can be much more than 100%.

### Synchronized haustral pressure waves (SHPWs)

Rhythmic propagating contractions over 1 or 2 haustra at a characteristic frequency of ~3 cycles/min. The occurrence of this motor pattern is reported before and/or after a meal.

### Statistical analysis

Continuous variables were reported as mean ± standard deviation (SD), and categorical variables were reported as n and percentages. Continuous variables measured before and after the meal were compared using the Paired samples t test, for patients with both baseline and post-meal measurements. Continuous variables at baseline between subject groups were compared using independent t test. Multiple linear regression was used to compare post-meal continuous variables between subjects’ groups, adjusted for the baseline values; the post-meal value was the dependent variable, subjects group and baseline values were the independent variables. Proportions of subjects with HAPW or SHPW before and after meal were compared using McNemar’s test for patients with both baseline and post-meal measurement. Proportions of subjects with HAPW or SHPW at baselines between subject groups were compared using Fisher’s exact tests. Multiple logistic regression was used to compare the percentage of patients with HAPW or SHPW after meal between subjects’ group, adjusting for baseline data. Age was not included in the regression model because age was not significantly different between constipation and controls (45.2 vs 40.3, t = 1.4, df = 27, P = 0.18). All tests were two-sided with p values < 0.05 considered significant. Statistical analysis was performed with IBM SPSS Statistics for Windows, Version 22.0. Armonk, NY, USA.

The sample sizes of the two control groups were too small to do a regression comparison between patients with constipation and the two groups were combined for this analysis as shown in Table 1.

## Additional Information

**How to cite this article**: Chen, J.-H. *et al*. Intraluminal pressure patterns in the human colon assessed by high-resolution manometry. *Sci. Rep.* **7**, 41436; doi: 10.1038/srep41436 (2017).

**Publisher's note:** Springer Nature remains neutral with regard to jurisdictional claims in published maps and institutional affiliations.

## Supplementary Material

Supplementary Information

## Figures and Tables

**Table 1:** Pressure Patterns at Baseline and Response To Meal.

		Chronic Constipation (n = 15)		Controls (n = 14)	
		Baseline	Meal	Baseline	Meal
Simultaneous Pressure Waves (SPWs)	Occurrence (/30 min)	10.3 ± 7.7 (15)	13.9 ± 10.3 15)	14.7 ± 11.1 (14)	18.7 ± 17.2 (14)
	Maximum frequency (/min)	1.3 ± 0.4 (11)	1.7 ± 0.7 (11)	1.4 ± 0.6 (11)	1.9 ± 0.7 (11)*
	Average Pressure duration (s)	15.2 ± 5.4 (14)	14.4 ± 5.4 (13)	15.5 ± 3.5 (13)	13.7 ± 2.7 (13)
	Propagation distance (cm)	33.8 ± 3.0 (14)	29.6 ± 6.4 (13)**	32.3 ± 6.5 (13)	29.8 ± 7.1 (13)
	Average amplitude (mmHg)	14.2 ± 5.6 (14)	12.5 ± 5.4 (13)	14.6 ± 6.7 (14)	14.6 ± 11.1 (14)
Antegrade Pressure waves (APWs)	Occurrence (/30 min)	0.4 ± 0.8 (15)	1.5 ± 3.0 (15)	0.9 ± 1.7 (13)	1.6 ± 2.6 (14)
	Maximum frequency (/min)	(0)	1.6 ± 1.3 (2)	2.0 (1)	2.2 ± 1.0 (3)
	Average Pressure duration (s)	15.3 ± 3.5 (4)	17.8 ± 3.4 (6)	16.1 ± 7.3 (4)	14.1 ± 4.5 (7)
	Propagation distance (cm)	21.3 ± 10.8 (4)	26.4 ± 11.2 (6)	29.3 ± 6.2 (5)	30.2 ± 6.3 (8)
	Average amplitude (mmHg)	19.6 ± 22.4 (4)	22.0 ± 10.3 (6)	20.5 ± 9.2 (5)	18.6 ± 8.8 (8)
Retrograde Pressure Waves (RPWs)	Occurrence (/30 min)	0.1 ± 0.3 (15)	0.7 ± 1.6(15)	0.6 ± 1.9 (14)	0.7 ± 1.4 (14)
	Maximum frequency (/min)	(0)	(0)	0.4 (1)	(0)
	Average Pressure duration (s)	15.0 (1)	11.7 ± 4.1 (4)	21.9 ± 7.2 (2)	16.0 ± 5.3 (5)
	Propagation distance (cm)	18.0 (1)	19.9 ± 8.3 (4)	26.0 ± 14.1(2)	24.9 ± 10.6 (5)
	Average amplitude (mmHg)	12.0 (1)	26.6 ± 9.1 (4)	15.8 ± 14.3 (3)	17.6 ± 7.5 (6)
Isolated Pressure Transients	Maximum amplitude (mmHg)	132.7 ± 53.8 (15)	138.4 ± 44.9 (15)	113.4 ± 51.7 (14)	108.8 ± 47.1 (13)
	Percentage of sensors involved	36.4 ± 15.0 (15)	39.3 ± 17.2 (15)	37.6 ± 15.2 (14)	39.0 ± 15.2 (14)
	Percentage of time involved	70.1 ± 18.1 (15)	68.6 ± 14.3 (15)	69.4 ± 16.4 (13)	62.7 ± 19.9 (13)
	Percentage with amplitude < 50 mmHg	97.6 ± 8.6 (15)	99.3 ± 1.5 (15)	99.97 ± 0.1 (14)	99.6 ± 1.4 (14)
	Percentage with amplitude 50–100 mmHg	49.7 ± 22.5 (15)	55.0 ± 23.8 (15)	37.8 ± 30.3 (14)	32.3 ± 22.6 (14)^#^
	Percentage with amplitude > 100 mmHg	21.5 ± 16.4 (15)	22.6 ± 15.7 (15)	15.6 ± 13.5 (14)	12.3 ± 12.1 (14)
High amplitude pressure waves (HAPWs)	Occurrence (%)	1 (7.1%) (15)	2 (14.3%) (15)	0 (0%) (14)	2 (14.3%) (14)
Synchronized haustral pressure waves	Occurrence (%)	1 (7.1%) (15)	9 (64.2%) 15)***	1 (7.7%) (14)	4 (30.8%) (14)

Values are expressed as mean ± SD.

* P = 0.027 (t = −2.6, df = 9), **P = 0.024 (t = 2.6, df = 11), before and after meal, same subject group (paired samples t test). ^#^P = 0.025 (B = −15.4, 95% CI −2.1 to −28.8) for post meal in constipation vs post meal in control (Multiple linear regression, adjusted for baseline values). ***P = 0.008 before and after meal, using McNemar’s test, same subject group. No statistical analysis was performed when n =< 5. Synchronized haustral pressure waves always alternated with segmental haustral activity.
