# Supplementary material for: Intraluminal pressure patterns in the human colon assessed by high-resolution manometry
Source: Sci Rep. 2017 Feb 20;7:41436. doi: 10.1038/srep41436 (PMC5316981; doi:10.1038/srep41436)
Supplement: Supplementary Information [file srep41436-s1.pdf]

ONLINE SUPPORT FOR: "INTRALUMINAL PRESSURE PATTERNS IN THE HUMAN COLON ASSESSED BY HIGH-RESOLUTION MANOMETRY"

Ji-Hong Chen (1,2), Yuanjie Yu (1), Zixian Yang (1), Wen-Zhen Yu (1), Wu Lan Chen (2,3), Hui Yu (2,3), Marie Jeong-Min Kim (2,4), Min Huang (1), Shiyun Tan (1), Hesheng Luo (1), Jianfeng Chen (5), Jiande DZ Chen (5,6) and Jan D. Huizinga (2,1).

1. Renmin Hospital of Wuhan University, Key Laboratory of Hubei Province for Digestive System Diseases, Wuhan, Hubei, China
2. McMaster University, Farncombe Family Digestive Health Research Institute, Department of Medicine, division of Gastroenterology, Hamilton, Ontario, Canada
3. Honours Biology Pharmacology Coop Program, McMaster University
4. Honours Health Sciences Program, McMaster University
5. MedKinetic Inc., Ningbo, China
6. Johns Hopkins University School of Medicine, Baltimore, USA

TABLE 1S -- PRESSURE PATTERNS AT BASELINE AND RESPONSE TO MEAL IN THE TWO CONTROL GROUPS

		Patients controls (n=9)		Healthy volunteers (n=5)	
		Baseline	Meal	Baseline	Meal
Simultaneous Pressure Waves (SPWs)	Occurrence (/30 min)	17.1±13.1 (9)	22.9 ±20.2 (9)	10.2 ± 4.9 (5)	11.1 ± 5.7 (5)
	Maximum rhythmicity (/min)	1.4 ± 0.6 (8)	2.0 ± 0.7 (7)	1.2 ± 0.2 (3)	1.7 ± 0.9 (4)
	Average Pressure duration (s)	16.0 ± 3.4 (8)	13.5 ± 3.1 (8)*	14.6 ± 4.0 (5)	14.0 ± 2.2 (5)
	Propagation distance (cm)	31.3 ± 8.1 (8)	29.0 ± 7.3 (8)	34.0 ± 2.5 (5)	31.2 ± 7.3 (5)
	Average amplitude (mmHg)	15.2 ± 7.7 (9)	18.0 ± 12.4 (9)	13.7 ± 5.1 (5)	8.5 ± 4.5 (5)** #
Antegrade Pressure waves (APWs)	Occurrence (/30 min)	1.1 ± 1.9 (8)	1.1 ± 1.7 (9)	0.8 ± 1.4 (5)	2.6 ± 3.7 (5)
	Maximum rhythmicity (/min)	2.0 (1)	2.8 (0.4) (2)	(0)	1.0 (1)
	Average Pressure duration (s)	18.6 ± 6.4 (3)	13.0 ± 4.8 (5)	8.5 (1)	16.9 ± 3.0 (2)

	Propagation distance (cm)	28.9 ± 6.2 (3)	30.7± 5.9 (5)	30.0 ± 8.5 (2)	29.4 ± 8.4 (3)
	Average amplitude (mmHg)	15.1±2.2 (3)	15.8 ± 5.4 (5)	28.5 ± 10.6 (2)	23.3 ± 12.8 (3)
Retrograde Pressure Waves (RPWs)	Occurrence (/30 min)	0.1 ± 0.3 (9)	0.2 ± 0.5 (9)	1.4 ± 3.1 (5)	1.4 ± 2.1 (5)
	Maximum rhythmicity (/min)	(0)	(0)	0.4 (1)	(0)
	Average Pressure duration (s)	21.9 ± 7.2 (2)	14.1 ±5.0 (3)	(0)	18.9 ± 5.8 (2)
	Propagation distance (cm)	26.0 ± 14.1 (2)	22.3 ± 12.2 (3)	(0)	28.8 ± 10.3 (2)
	Average amplitude (mmHg)	7.7 ± 3.7 (2)	15.9 ± 2.9 (3)	32.0 (1)	19.2 ± 11.1 (3)
Isolated Pressure Transients	Maximum amplitude (mmHg)	115.6 ± 42.5 (9)	109.0 ± 56.4 (9)	109.4 ± 71.1 (5)	108.3 ± 19.2 (4)
	Percentage of sensors involved	36.3 ±18.1 (9)	35.6 ± 15.0 (9)	40.0 ± 9.0 (5)	45.2 ± 15.0 (5)
	Percentage of time involved	71.2 ± 17.8 (9)	60.2 ± 23.3 (9)	65.5± 14.1 (4)	68.5 ± 9.1 (4)
	Percentage with amplitude <50 mmHg	100 ± 0 (9)	100 ± 0 (9)	99.9 ± 0.1 (5)	98.9 ± 2.4 (5)
	Percentage with amplitude 50-100 mmHg	43.4 ± 33.6 (9)	38.2 ±25.2	27.8 ± 22.9 (5)	21.6 ± 13.1 (5)
	Percentage with amplitude > 100 mmHg	17.4 ± 13.0 (9)	16.7 ± 12.9 (9)	12.3 ± 15.3 (5)	4.3 ± 4.2 (5)
High Amplitude Pressure Waves (HAPWs)	Occurrence (%)	0 (0%) (9)	2 (22.2%) (9)	0 (0%) (5)	0 (0%) (5)
Synchronized Haustral Pressure Waves	Occurrence (%)	1 (11.1%) (9)	2 (22.2%) (9)	1 (20%) (5)	2 (40%) (5)

* p=0.018 (t=3.1, df=7), **p=0.007 (t=5.2, df=4), baseline vs after meal, same subject group (paired samples t test)
# p=0.043 (B=-7.5, 95% CI -0.3 to -14.8) for post meal values in patients controls vs healthy controls (Multiple linear regression, adjusted for baseline values). No statistics were done with n<5.

TABLE 2S -- MOTOR PATTERNS AT BASELINE AND RESPONSE TO MEAL

PATIENTS WITH CONSTIPATION, WITH AND WITHOUT RESPONSE TO TREATMENT

		Constipation Responders (n=8)		Constipation Non Responders (n=3)	
Mean (min-max) (n)		Baseline	Meal	Baseline	Meal
Simultaneous Pressure Waves	Occurrence (/30 min)	12.5 (5.0-21.0) (8)	18.5 (8.5-36.6) (8)	5.3 (0.5-14.5) (3)	8.3 (0-16.0) (3)
	Maximum rhythmicity (/min)	1.3 (0.8-2.2) (8)	2.1 (1.5-3.0) (7)	1.3 (1)	0.6 (0.3-0.9) (2)
	Average Pressure duration (s)	16.5 (9.1-24.0) (8)	13.6 (10.4-20.6) (8)	12.8 (3.2-20.0)(3)	18.5 (18.3-18.7) (2)
	Propagation distance (cm)	33.4 (29.0-36.0) (8)	27.6 (20.0-36.0) (8)	35.2 (33.5-36.0) (3)	35.1 (34.2-36.0) (2)
	Average amplitude (mmHg)	14.4 (7.2-23.4)(8)	13.0 (6.4-21.9) (8)	15.0 (8.7-20.0) (3)	15.3 (11.5-19.0) (2)
Antegrade Pressure waves	Occurrence (/30 min)	0.4 (0-1.4) (8)	2.2 (0-10.3) (8)	0.9 (0-2.6) (3)	1.8 (0-5.4) (3)
	Maximum rhythmicity (/min)	(0)	1.6 (0.7-2.5) (2)	(0)	(0)
	Average Pressure duration (s)	14.7 (10.0-17.0)(3)	17.2 (14.9-23.0) (5)	17.1 (1)	21.1 (1)
	Propagation distance (cm)	16.4 (10.0-20.0) (3)	24.5 (10.0-36.0) (5)	36.0 (1)	36.0 (1)
	Average amplitude (mmHg)	22.3 (5.0-53.0) (3)	23.1 (6.0-35.0) (5)	11.5 (1)	16.5 (1)
Retrograde Pressure Waves	Occurrence (/30 min)	0.2 (0-1.2) (8)	1.3 (0-5.0) (8)	0(3)	0(3)
	Maximum rhythmicity (/min)	0	0	(0)	(0)
	Average Pressure duration (s)	15.0 (1)	11.7 (6.0-15.0) (4)	(0)	(0)
	Propagation distance (cm)	18.0 (1)	19.9 (10.0-30.3) (4)	(0)	(0)
	Average amplitude (mmHg)	12.0 (1)	26.6 (15.2-36.0) (4)	(0)	(0)
Isolated	Maximum amplitude (mmHg)	147.3 (60.0-212.0)	142.1 (45.0-223.0)	110.0 (47.0-149.0) (3)	153.0 (92.0-191.0) (3)

Pressure Transients		(8)	(8)		
	Percentage of sensors involved	45.1 (23.0-63.0) (8)	49.9 (23.0-76.0) (8)	22.4 (11.0-32.0) (3)	29.9 (18.0-36.0) (3)
	Percentage of time involved	76.5 (54.0-95.0) (8)	74.2 (62.0-85.0) (8)	61.2 (35.0-94.0 (3)	67.1 (41.8-88.0) (3)
	Percentage with amplitude <50 mmHg	100.0 (100.0-100.0)(8)	99.2 (96.0-100.0) (8)	88.2 (66.7-100.0) (3)	100.0 (100.0-100.0)(3)
	Percentage with amplitude 50-100 mmHg	53.1 (20.0-80.7) (8)	57.0 (7.5-83.7) (8)	43.6 (35.0-48.0) (3)	36.5 (15.5-67.0) (3)
	Percentage with amplitude > 100 mmHg	25.2 (0-47.0) (8)	23.8 (0-40.0) (8)	11.4 (0-24.0) (3)	20.2 (0-42.0) (3)
High Amplitude Pressure Waves (HAPWs)	Occurrence (%)	1 (12.5%) (8)	1 (12.5%) (8)	0(0%) (2)	0(0%) (2)
Synchronized Haustral Pressure Waves	Occurrence (%)	0 (0%) (8)	6 (75.0%) (8)	0(0%) (2)	0(0%) (2)

No statistical comparisons were performed because of low sample size of non-responders. Four of the 15 patients were not available for follow up.
